# Cross-sectional and longitudinal association of seven DNAm-based predictors with metabolic syndrome and type 2 diabetes

**DOI:** 10.1186/s13148-025-01862-8

**Published:** 2025-04-08

**Authors:** Suet Mei Chew, Alexander Teumer, Pamela R. Matías‑García, Christian Gieger, Juliane Winckelmann, Karsten Suhre, Christian Herder, Wolfgang Rathmann, Annette Peters, Melanie Waldenberger

**Affiliations:** 1 Research Unit Molecular Epidemiology, Institute of Epidemiology, Helmholtz Zentrum München, German Research Center for Environmental Health, Neuherberg, Germany; 2 Department of Medical Information Processing, Biometry and Epidemiology (IBE), Ludwig-Maximilians-Universität München, Munich, Germany; 3 Department of Psychiatry and Psychotherapy, University Medicine Greifswald, Greifswald, Germany; 4 German Center for Cardiovascular Research (DZHK), Partner Site Greifswald, Greifswald, Germany; 5 German Center for Cardiovascular Research (DZHK), Partner Site Munich Heart Alliance, Munich, Germany; 6 Institute of Neurogenomics, Helmholtz Zentrum München, German Research Center for Environmental Health, Neuherberg, Germany; 7 Institute of Human Genetics, Technische Universität München, Munich, Germany; 8 Chair of Neurogenetics, Technische Universität München, Munich, Germany; 9 Bioinformatics Core, Weill Cornell Medicine-Qatar, Education City, Doha, Qatar; 10 Department of Physiology and Biophysics, Weill Cornell Medicine, New York, NY USA; 11 Englander Institute for Precision Medicine, Weill Cornell Medicine, New York, NY USA; 12 Institute for Clinical Diabetology, German Diabetes Center, Leibniz Center for Diabetes Research at Heinrich Heine University Düsseldorf, Düsseldorf, Germany; 13 Department of Endocrinology and Diabetology, Medical Faculty and University Hospital Düsseldorf, Heinrich Heine University Düsseldorf, Düsseldorf, Germany; 14 German Center for Diabetes Research (DZD), Neuherberg, Germany; 15 Institute of Biometrics and Epidemiology, German Diabetes Center, Leibniz Center for Diabetes Research at Heinrich-Heine-University Düsseldorf, Düsseldorf, Germany; 16 Institute of Epidemiology, Helmholtz Zentrum München, German Research Center for Environmental Health, Neuherberg, Germany

**Keywords:** Epigenetic age acceleration, DNAm age, Metabolic syndrome, Diabetes, Risk prediction

## Abstract

**Background:**

To date, various epigenetic clocks have been constructed to estimate biological age, most commonly using DNA methylation (DNAm). These include “first-generation” clocks such as DNAmAgeHorvath and “second-generation” clocks such as DNAmPhenoAge and DNAmGrimAge. The divergence of one’s predicted DNAm age from chronological age, termed DNAmAge acceleration (AA), has been linked to mortality and various aging-related conditions, albeit with varying findings. In metabolic syndrome (MetS) and type 2 diabetes (T2D), it remains inconclusive which DNAm-based predictor(s) is/are closely related to these two metabolic conditions. Therefore, we examined the cross-sectional associations between seven DNAm-based predictors and prevalent metabolic conditions in participants with methylation data from the KORA study. We also analyzed the longitudinal association with time-to-incident T2D and the relative prognostic value compared to clinical predictors from the Framingham 8-year T2D risk function in predicting incident disease over eight years.

**Results:**

GrimAA and PhenoAA difference demonstrated consistently significant associations in the cross-sectional and longitudinal analyses. GrimAA difference reported a larger effect: with prevalent MetS at F4 (odds ratio = 1.09, 95% confidence interval = [1.06–1.13], *p* = 2.04E–08), with prevalent T2D at F4 (odds ratio = 1.09 [1.04–1.13], *p* = 1.38E–04) and with time-to-incident T2D (hazards ratio = 1.05 [1.01–1.10], *p* = 0.02) for each year increase in GrimAA difference. Mortality risk score was significantly associated with both prevalent metabolic conditions but not in the longitudinal analysis. The inclusion of DNAm-based predictor in the model with Framingham clinical predictors improved discriminative ability, albeit not significantly. Notably, the DNAm-based predictor, when fitted separately, showed a discriminative ability comparable to that of the model with clinical predictors. Overall, no clear pattern of significant associations was identified in the epigenetic measures from the “first-generation” clocks.

**Conclusions:**

GrimAA, PhenoAA difference and mortality risk score, derived from the “second-generation” clocks, demonstrated significant associations with both MetS and T2D. These DNAm-based predictors may be useful biomarkers for risk stratification and disease prognosis in our study sample of European ancestry. Further research is warranted to investigate the generalizability of our findings across different ancestries and to examine the underlying shared biological mechanisms.

**Supplementary Information:**

The online version contains supplementary material available at 10.1186/s13148-025-01862-8.

## Background

Chronological age stands as a major risk factor common to a plethora of chronic (non-communicable) diseases, ranging from neurodegenerative to metabolic disorders and cancer. While chronological age progresses at a constant rate, the marked interindividual variation in health outcomes observed between individuals of the same age could be partly attributed to the varying rates of biological aging, a state known as accelerated or decelerated aging [1]. Chronic diseases can represent a manifestation of accelerated aging—that is, when biological age exceeds chronological age—as a result of a complex interplay between genetics, lifestyle factors, environmental and social determinants of health [1].

To date, multiple estimators have been established to measure biological age, out of which epigenetic clocks are, by far, the most promising biomarkers, built based on DNA methylation (DNAm) [2, 3]. DNAm is the most extensively studied epigenetic process, whereby a methyl group is attached at or removed from a DNA nucleotide, typically at the cytosine in the cytosine-guanine dinucleotides (CpG) [4].

Several epigenetic clocks have been developed by quantitatively combining the DNAm levels from sets of CpGs into a composite predictor. The initial clocks were aimed in predicting chronological age (termed collectively as “first-generation” epigenetic clocks), out of which the two most recognized clocks are by Horvath (DNAmAgeHorvath) [5], and Hannum (DNAmAgeHannum) [6]. Age acceleration (AA) is then derived from the difference between the estimated biological age and chronological age to reflect the state of accelerated aging or decelerated aging (i.e., positive or negative AA difference) [2]. Both epigenetic measures were then refined to be reflective of specific aging aspects: (i) intrinsic epigenetic age acceleration (IEAA) as residuals of DNAmAgeHorvath, which mirrored intrinsic aging independent of leukocytes composition [7]; and (ii) extrinsic epigenetic age acceleration (EEAA) as residuals of DNAmAgeHannum, which specifically reflected aging of the immune system [8].

Subsequently, a “second-generation” of epigenetic clocks were developed using age-related outcomes as training phenotype. The DNAmPhenoAge clock was trained to reflect physiological dysregulation through age and nine biological biomarkers [9]. DNAmGrimAge, as the name suggests, was trained to predict mortality using well-established correlates of morbidity or mortality, which included seven DNAm-based surrogates of plasma proteins and one of smoking pack-years [10]. Another similar DNAm-based predictor, mortality risk score (MRS) was trained to predict all-cause mortality. However, it differed from the aforementioned predictors, in that MRS was not expressed in the unit of years but as the sum of methylation levels of 10 CpGs [11].

To unravel the role of biological aging in the interindividual heterogeneity in health outcomes, numerous studies have highlighted DNAmAge or AA being closely linked to differential susceptibility to death and a myriad of aging-related conditions: all-cause mortality [8, 12–15], cognitive and functional decline [15], cardiovascular disease [14, 16] and many more [2]. Nonetheless, overall findings vary with differential association observed for each epigenetic measure with various traits/diseases, presumably attributable to the lack of CpG overlap between the measures and, consequently, distinctive aging pathways underlying each [17]. For metabolic conditions such as metabolic syndrome (MetS) and type 2 diabetes (T2D), it has not been clearly established which epigenetic measure(s) is/are most/more related to the conditions.

MetS is a constellation of cardio-metabolic risk factors, including increased waist circumference, increased blood pressure (BP), hyperglycaemia, hypertriglyceridaemia, and reduced high-density lipoprotein cholesterol (HDL-C) [18]. MetS can predispose one to metabolic diseases, such as T2D and cardiovascular diseases [19]. The development of these metabolic conditions indicates an alternative aging trajectory, characterized by accelerated aging and shared characteristics, such as epigenetic alterations, leading to cellular senescence and inflammation, especially in adipose tissue [20]. With DNAm implicated in the pathological process of metabolic traits/diseases [20], multiple studies have examined the underlying relationship. Nannini et al. found higher MetS score to be associated with accelerated IEAA and EEAA in young adults in the USA [21]. In a study sample of Korean ancestry, MetS score was identified to be positively associated with only the GrimAge clock in middle-age adults, among the other clocks [22]. Notwithstanding the varying findings, existing evidence highlights AA as a potential biomarker of metabolic conditions. Some studies focused only on one or two specific epigenetic measures, while some examined only a specific subgroup from the general population [16, 21–26]. In this study, we evaluated and compared the utility of seven DNAm-based predictors (HorvathAA, GrimAA, PhenoAA, HannumAA, EEAA, IEAA and MRS), in their association with prevalent MetS and T2D, in a large sample of European ancestry from the KORA (Kooperative Gesundheitsforschung in der Region Augsburg) study. We also examined the longitudinal association with time-to-incidence of T2D and the prognostic value of the biomarker in predicting 8-year T2D incidence risk.

## Methods

### Study population

The study population comprised participants from the KORA study, a research platform which has been conducting health surveys in the population with German nationality living in the region of Augsburg in southern Germany [27]. Our study cohort originated from the S4 survey (1999–2001), one of the four KORA cross-sectional surveys [27]. In the baseline S4 survey, 4261 participants aged 25–74 years old were enrolled. To date, the S4 survey has two follow-up studies, namely F4 (2006–2008) and FF4 (2013–2014). In all three assessments, study participants completed a lifestyle questionnaire and underwent standardized medical examinations and biosamples collection, as described in detail elsewhere [27]. Additionally, the participants were followed up on their survival and morbidity status via a questionnaire until 2015–2016.

Of the 4261 participants at baseline, selection for methylation profiling was first done randomly among the participants who remained at F4, stratified by age categories with a higher representation of older participants, and conditioned upon consent for genetic data profiling. Profiling at S4 and FF4 was then performed for those selected at F4, resulting in a sample of 2661 participants who underwent methylation profiling at least at one of the timepoints. Subsequently, we excluded participants whose methylation data failed quality control or presented a mismatch in predicted sex.

Cross-sectional analyses of prevalent MetS and T2D were performed on the subsamples of participants with methylation data at F4 (*n* = 1722) and FF4 (*n* = 1872), respectively. The methylation data at S4 was not used as MetS could not be determined for participants aged < 55 without fasting glucose and/or lipid measurements. Conversely, longitudinal analyses were conducted using the subsample of participants whose methylation data was profiled at baseline S4. For the Cox regression analysis of time-to-incident T2D, participants with confirmed or undetermined diabetes status at baseline S4 were excluded, resulting in a final subsample of 1456 participants (flowchart of participants in Additional file 1: Figure S1).

### Outcomes definition

Study outcomes included two metabolic conditions: MetS (as binary and ordinal variable) and T2D. MetS was defined based on the harmonized definition by Alberti et al. as the presence of at least three of the following five criteria: (1) waist circumference ≥ 94 cm in men or ≥ 80 cm in women; (2) fasting serum triglycerides ≥ 150 mg/dl and/or drug treatment for elevated triglycerides; (3) serum HDL-C < 40 mg/dl in men or < 50 mg/dl in women and/or drug treatment to reduce HDL (fibrates); (4) systolic BP ≥ 130 mmHg or diastolic BP ≥ 85 mmHg and/or intake of antihypertensive medication; (5) fasting serum glucose level ≥ 100 mg/dl and/or intake of antidiabetic medication [18]. MetS score was defined as the summation of the number of MetS components present based on the above criteria, with a range of 0–5.

T2D was determined based on participants’ self-report of being clinically diagnosed with diabetes or intake of glucose-lowering medication, which was subsequently validated with the attending physician or medical records. Time-to-incident T2D was calculated as the difference in years between baseline age and the age at T2D diagnosis. Participants who did not develop T2D or were lost to follow-up by the end of the observation period (i.e., in 2015 or 2016) were right-censored, with survival time calculated as the difference in years between baseline age and the age reported at the last follow-up. Additionally, participants diagnosed with other types of diabetes were censored at the age of their diagnosis.

### DNA methylation profiling

DNA methylation was measured in whole blood using the Illumina Infinium HumanMethylation 450K at S4 and F4, and EPIC BeadChip at FF4. At S4 and F4, the normalization of methylation data was performed according to the CPACOR pipeline [28], starting with the exclusion of the 65 single-nucleotide polymorphism markers, background correction using the R package *minfi* [29], and subsequently setting probes to missing if the signals had a detection *p*-value of > 0.01 or were summarized from ≤ 3 functional beads. Thereafter, samples with a detection rate of ≤ 95% were excluded. With the remaining samples, quantile normalization was performed on the signal intensity values, divided into six categories by probe type and color channel. After these preprocessing steps, methylation data was then used for the computation of epigenetic age estimates. Methylation data at FF4 underwent similar preprocessing procedures, including background correction, removal of probes of which the cross-reactive probes being specific to the EPIC array, sample filtering and quantile normalization.

### Computation of DNAm-based predictors

We evaluated a total of seven epigenetic age measures, namely HorvathAA, HannumAA, GrimAA, PhenoAA, EEAA, IEAA and MRS (further details of each measure are provided in Additional file 2: Table S7). The first four measures were calculated using estimates obtained from the online DNAmAge clock (https://dnamage.genetics.ucla.edu/) [5] under the advanced analysis option: DNAmAgeHorvath [5], DNAmAgeHannum [6], DNAmPhenoAge [10] and DNAmGrimAge [11]. Age acceleration was then derived as the difference between the predicted DNAmAge and chronological age. We defined it as difference instead of residual of DNAmAge regressed on chronological age, as AA difference is more intuitive for interpretation and represents as an individual parameter, while residual has a mean of zero and reflects as a population parameter.

Two of the clocks, DNAmAgeHannum and DNAmAgeHorvath, generated the raw residuals of EEAA and IEAA, respectively. IEAA was derived by regressing DNAmAgeHorvath on chronological age and blood immune cells counts. DNAmAgeHannum was further transformed by up-weighting the contribution of three age-related blood cell types to produce EEAA [8]. The last measure, MRS, is an epigenetic clock developed by Zhang et al., derived as the sum of the individual methylation *β* values of ten CpGs multiplied by their respective coefficients [11]. While MRS was originally constructed using Illumina 450K array, we calculated a “modified” version of MRS as two out of the ten CpGs were unavailable in the EPIC BeadChip used at FF4. This allowed us to compare its utility with the modified version.

If GrimAA reported a significant association with either of the two metabolic conditions, we further analyzed its eight underlying components (adrenomedullin, beta-2 microglobulin, cystatin C, growth differentiation factor 15, leptin, plasminogen activation inhibitor 1, tissue inhibitor metalloproteinase 1, smoking pack-years) [10], to identify the component(s) driving the association.

### Statistical analysis

In the cross-sectional analyses of prevalent MetS and T2D, as well as the Cox regression of time-to-T2D, we included chronological age and sex in the crude model, while the fully adjusted model accounted additionally for smoking status (current/ever/never), alcohol consumption (g/day), body mass index (kg/m^2^, only for T2D), and physical activity level (active/inactive). Physical activity was defined based on the answer given to how many hours weekly were spent doing sport both in winter and summer: (i) > 2 h regularly, (ii) approximately 1 h regularly, (iii) approximately 1 h irregularly, or (iv) almost none/none.

We adjusted for chronological age in all models due to its known effects on morbidity and mortality. Notably, IEAA and EEAA were residuals derived from regressing DNAmAge on chronological age and were, therefore, mathematically uncorrelated with age. As for the other four AA measures (i.e. HorvathAA, HannumAA, GrimAA and PhenoAA difference), there might still be correlation with age in our dataset. Nonetheless, adjusting for age in the regression models could reduce variation of the outcome or residual correlation with age independent of the DNAmAge, potentially increasing statistical power to detect significant associations. Additionally, to eliminate the technical effects of methylation data, we included the first 20 principal components (PCs) of the positive control probes and a batch variable (only for S4 methylation data, defined as 1 for the 86 S4 samples processed separately and 0 for all the others). All covariates for adjustment were the same for all DNAm-based predictors, except for DNAm-predicted pack years (one of the eight GrimAge components) with the covariate of smoking status excluded.

Adjusting for the aforementioned covariates, we conducted the cross-sectional analyses using logistic regression, modeling the respective metabolic conditions (i.e., prevalent MetS and T2D) modeled as outcome, and each of the seven DNAm-based predictor as the explanatory variable. Apart from MetS as a binary outcome, we examined the ordinal outcome of MetS score using ordinal regression with the function *polr* in the R package *MASS*. The proportional odds assumption was checked using the Brant test in the R package *Brant* [30]. We analyzed using data from F4 and FF4 and compared the results to assess whether the patterns of significant associations remained consistent.

For the longitudinal analyses, we used the Cox proportional hazards regression, modeling time-to-incidence of T2D as outcome and each DNAm-based predictor as the explanatory variable. The proportional hazards assumption was examined using a statistical test and graphical diagnostics based on the Schoenfeld residuals. For all the analyses, the *p*-value was set at 0.05, no adjustment for multiple testing was done considering the exploratory nature of the analyses. To identify the concordantly significant DNAm-based predictor(s), we used the results of the fully adjusted model. Of note, the coefficient of MRS was standardized to its standard deviation to enable comparison of effect sizes across the DNAm-based predictors, as MRS was not constructed as an AA measure.

In sensitivity analyses, we removed outliers for each DNAm-based predictor with values beyond ± 1.5 interquartile range (IQR) of the 25% and 75% quartile and repeated the analyses using the fully adjusted model for each outcome. For the four AA difference measures, we adjusted for the following imputed leukocyte count, in addition to the covariates in the fully adjusted model: naïve CD8 + T, exhausted cytotoxic CD8 + T cells, plasma blasts, CD4 + T, natural killer cells, monocytes, and granulocytes. Although these four DNAm-based predictors are regarded as extrinsic AA measures, this sensitivity analysis sought to identify any attenuation of effect size after adjusting for these age-related cell types. As a secondary analysis of GrimAA, we further analyzed its eight underlying components to identify the one driving its association with MetS and/or T2D, in the event that GrimAA was significantly associated with the prevalent metabolic conditions.

Lastly, we specifically conducted the area under the curve (AUC) analyses on the DNAm-based predictor(s) identified as concordantly significant in the preceding analyses, exploring the prognostic value for incidence of T2D over an 8-year period. We compared the discriminative ability between the model with established clinical predictors from the Framingham 8-Year risk of T2D algorithm and the model with DNAm-based predictors. The Framingham clinical predictors include: (i) fasting glucose level of 100–126 mg/dL, (ii) body mass index (BMI) categories of 25.0–29.9 kg/m^2^ or ≥ 30 kg/m^2^, (iii) BP ≥ 130/85 mmHg or receiving drug treatment, (iv) HDL-C level of < 40 mg/dl in men or ≥ 50 mg/dl in women, (v) parental history of diabetes mellitus, and (vi) triglyceride level of ≥ 150 mg/dL [31]. In the model with DNAm-based predictors, we adjusted for baseline age, sex and technical effects, which included the first five PCs and a batch variable. For each model, we plotted the ROC curves and assessed the discriminative ability of the DNAm-based predictor(s) to classify risk using the C statistic, represented by the AUC.

## Results

### Sample characteristics

The study sample comprised (i) 1530 participants from KORA S4 whose methylation data was profiled (used in longitudinal analyses), and (ii) 1722 and 1918 participants profiled at KORA F4 and FF4, respectively (used in cross-sectional analyses). Additionally, the final sample was selected after excluding participants with methylation data which failed the quality control criteria or indicated a sex mismatch (see Additional file 1: Figure S1 for flowchart of the participants).

Table 1 presents the summary characteristics of the longitudinal study sample from KORA S4, as well as the comparison between participants who were followed up until FF4 versus those lost to follow-up. Overall, at baseline (S4), the majority were female (50.6%) with mean age of 54.0 years (standard deviation, SD = 8.9). T2D prevalence was 7.2%, while almost half (49.4%) of participants aged ≥ 55 years had MetS (i.e., presence of at least three of the five MetS components).

**Table 1:** Baseline characteristics of study sample from KORA S4 and comparison between participants who completed follow-up at FF4 and participants lost to follow-up

Baseline characteristics	Study sample from KORA S4 with baseline epigenetic measures					
	Overall	Followed up until FF4		Lost to follow-up		*p* value
N	1530	1085		445		–
Male, *n* (%)	756 (49.4)	540 (49.8)		216 (48.5)		0.703
Age, mean (SD), years	54.0 (8.9)	52.8 (8.7)		56.8 (8.8)		< 0.001^*^
HorvathAA difference, mean (SD), years	1.1 (4.9)	1.2 (4.8)		0.8 (5.2)		0.103
Positive HorvathAA difference, *n* (%)	907 (59.3)	659 (60.7)		248 (55.7)		0.080
HannumAA difference, mean (SD), years	4.6 (5.3)	4.6 (5.2)		4.6 (5.5)		0.845
Positive HannumAA difference, *n* (%)	1282 (83.8)	910 (83.9)		372 (83.6)		0.955
PhenoAA difference, mean (SD), years	− 4.6 (6.6)	− 4.7 (6.4)		− 4.3 (6.9)		0.369
Positive PhenoAA difference, *n* (%)	341 (22.3)	237 (21.8)		104 (23.4)		0.559
GrimAA difference, mean (SD), years	1.8 (5.2)	1.7 (5.0)		2.1 (5.7)		0.095
Positive GrimAA difference, *n* (%)	910 (59.5)	654 (60.3)		256 (57.5)		0.349
Positive EEAA, *n* (%)	780 (51.0)	524 (48.3)		256 (57.5)		0.001^*^
Positive IEAA, *n* (%)	769 (50.3)	532 (49.0)		237 (53.3)		0.148
Mortality risk score, mean (SD), methylation *β* value^a^	− 2.7 (0.5)	− 2.7 (0.4)		− 2.6 (0.5)		< 0.001^*^
Mortality risk score risk level, *n* (%)^a^						
Low	554 (36.2)	426 (39.3)		128 (28.8)		< 0.001^*^
Moderate	836 (54.6)	574 (52.9)		262 (58.9)		
High	98 (6.4)	51 (4.7)		47 (10.6)		
BMI, mean (SD), kg/m^2a^	27.7 (4.5)	27.4 (4.3)		28.5 (4.8)		< 0.001^*^
Alcohol consumption, median (IQR), g/day^a^	8.4 (0.9–25.7)	8.6 (2.9–25.7)		5.7 (0.0–25.7)		0.012^*^
Hypertension, *n* (%)^a^	642 (42.0)	424 (39.1)		218 (49.0)		0.002^*^
Physical activity, *n* (%)^a^						< 0.001^*^
Inactive	772 (50.5)	501 (46.2)		271 (60.9)		
Active	750 (49.0)	579 (53.4)		171 (38.4)		
Smoking status, *n* (%)^a^						0.125
Never	649 (42.4)	463 (42.7)		186 (41.8)		
Former	568 (37.1)	416 (38.3)		152 (34.2)		
Current	311 (20.3)	205 (18.9)		106 (23.8)		
Diabetes, *n* (%)						
Type 2	110 (7.2)	57 (5.3)		53 (11.9)		< 0.001^*^
No diabetes	1417 (92.6)	1025 (94.5)		392 (88.1)		
Other diabetes types	3 (0.2)	3 (0.2)		0 (0.0)		

MetS baseline measurement	Sample subset of participants aged ≥ 55 years at S4^b^			
	Overall	Followed up until FF4	Lost to follow-up at FF4	*p* value
N	745	468	277	–
MetS, *n* (%)^c^	368 (49.4)	214 (45.7)	154 (55.6)	0.002*
MetS score, median (IQR)^c^	2.0 (1.0 – 3.0)	2.0 (1.0 – 3.0)	3.0 (2.0–3.0)	0.011^*^
Number of MetS component(s), *n* (%)^c^				0.008^*^
0 component, *n* (%)	39 (5.2)	27 (5.8)	12 (4.3)	
1 component, *n* (%)	138 (18.5)	98 (20.9)	40 (14.4)	
2 components, *n* (%)	157 (21.1)	107 (22.9)	50 (18.1)	
3 components, *n* (%)	195 (26.2)	115 (24.6)	80 (28.9)	
4 components, *n* (%)	96 (12.9)	62 (13.2)	34 (12.3)	
5 components, *n* (%)	35 (4.7)	17 (3.6)	18 (6.5)	

For continuous variables, *p*-value for equality between groups was determined by Student’s *t*-test (normal), by Mann–Whitney *U* test (nonnormal). For categorical variables, *p*-value for equality between groups was determined by chi-square test of independence. Any differences from 100% in the sum of percentage per category reflect missing values

SD, standard deviation; AA, age acceleration; EEAA, extrinsic epigenetic age acceleration; IEAA, intrinsic epigenetic age acceleration; IQR, interquartile range; BMI, body mass index; MetS, metabolic syndrome

^*^Statistically significant at *p* < 0.05

^a^Number of missing value(s) in the overall sample: 42 in MRS and risk level, 2 in BMI, 8 in alcohol consumption, 8 in hypertension, 8 in physical activity, and 2 in smoking

^b^At S4, MetS was only measured in participants who were aged 55 years and above

^c^Number of missing value(s) in the subsample aged ≥ 55 years: 37 in MetS, and 85 in MetS score and number of MetS component(s)

Relative to the individual chronological age, the overall mean HorvathAA difference was 1.1 years (SD 4.9), HannumAA difference 4.6 years (SD 5.3), PhenoAA difference − 4.6 years (SD 6.6) and GrimAA difference 1.8 years (SD 5.2). The values of the AA measures reflected some discrepancies, with the largest between HannumAA and PhenoAA difference. For example, based on HannumAA, participants were, on average, 4.6 years older than their chronological age, while PhenoAA indicated that they were 4.6 years younger. The majority (*n* = 836, 54.6%) had a moderate MRS risk level.

Of the 1530 S4 participants, 445 (29.1%) were lost to follow-up at FF4. Compared to those who remained until FF4, the former were older, as reflected by significantly higher mean chronological age (by 3.9 years). Additionally, there were significantly more participants with positive EEAA and higher MRS risk level. The prevalence of T2D, hypertension and MetS was also higher than that in participants who remained.

### Pairwise correlations between DNAm-based predictors

Additional file 1: Figure S2 and S3 present the scatterplot matrix displaying the correlations between chronological age and the respective DNAm-based measures. The four DNAmAge measures (namely DNAmAgeHorvath, DNAmAgeHannum, DNAmPhenoAge and DNAmGrimAge) showed moderate to strong positive correlation with chronological age and with each other (Pearson’s *r* = 0.7–0.8). While all the seven DNAm-based predictors displayed poor to almost zero correlation with chronological age, HorvathAA and GrimAA difference showed a fair but negative correlation (*r* =  − 0.4 for both). With each other, the predictors were not strongly correlated (*r* = 0.1–0.5) except for the pairs of Horvath AA difference with IEAA, and Hannum AA difference with EEAA. The strong correlations were expected as the respective pairs were derived from the same DNAmAge clock.

Upon examining the stability of the seven DNAm-based predictors longitudinally in the subsample with complete observations at S4, F4 and FF4 timepoints, all measures showed fair to moderate positive correlations across time, except for GrimAA difference reporting strong correlations in the range of 0.8 (Additional file 1: Figure S4).

### Cross-sectional analyses: MetS and T2D

Consistently in both F4 and FF4 subsample, we identified GrimAA difference, PhenoAA difference and MRS to be significantly associated with MetS, MetS score and T2 (Table 2). Of the three, GrimAA difference had the smallest p-value: An increase of one year corresponded to a 9.2% (95% CI = [5.9–12.6%]) increased odds of prevalent MetS (*p* = 2.0E–08) at F4, under the fully adjusted model (with comparable effect size in FF4). Expectedly, GrimAA difference showed the strongest statistical significance in association with MetS score (ordinal variable from 0 to 5) and T2D: Each additional year in GrimAA difference was associated with an 8.3% [5.9–11.1%] higher odds of a one-unit increase in MetS score (*p* = 6.9E–10) and 8.9% [4.2–13.7%] higher odds of prevalent T2D (*p* = 1.4E–04) at F4, under the fully adjusted model (with comparable effect size in FF4).

**Table 2:** Cross-sectional association between DNAm-based predictors and metabolic conditions

DNAm-based predictor	MetS in F4 subsample				MetS in FF4 subsample			
	OR	95% CI	*p*	*n*	OR	95% CI	*p*	n
HorvathAA	1.03	1.01, 1.06	**0.02**	1719	1.00	0.98, 1.03	0.67	1864
HannumAA	1.01	0.99, 1.03	0.32	1719	1.00	0.98, 1.04	0.47	1864
GrimAA	1.09	1.06, 1.13	**2.04E-08**	1719	1.08	1.05, 1.12	**4.80E-06**	1864
PhenoAA	1.03	1.01, 1.04	**4.67E-04**	1719	1.04	1.02, 1.06	**1.66E-04**	1864
IEAA	1.03	1.00, 1.05	**0.04**	1719	1.00	0.98, 1.03	0.91	1864
EEAA	1.01	1.00, 1.03	0.13	1719	1.01	0.99, 1.04	0.13	1864
MRS^a^	1.22	1.07, 1.39	**4.01E-03**	1468	–	–	–	–
MRS (modified)^a^	1.27	1.12, 1.43	**1.86E-04**	1674	1.15	1.01, 1.31	**0.03**	1864

DNAm-based predictor	MetS score in F4 subsample				MetS score in FF4 subsample			
	OR	95% CI	*p*	*n*	OR	95% CI	*p*	n
HorvathAA	1.02	1.00, 1.04	0.07	1708	1.02	1.00, 1.04	0.13	1852
HannumAA	1.01	0.99, 1.02	0.26	1708	1.00	0.98, 1.03	0.64	1852
GrimAA	1.08	1.06, 1.11	**6.87E-10**	1708	1.09	1.06, 1.12	**4.60E-10**	1852
PhenoAA	1.03	1.02, 1.04	**1.59E-06**	1708	1.04	1.02, 1.05	**3.64E-06**	1852
IEAA	1.01	0.99, 1.04	0.17	1708	1.01	0.99, 1.03	0.22	1852
EEAA	1.01	1.00, 1.03	0.06	1708	1.01	0.99, 1.03	0.23	1852
MRS^a^	1.17	1.05, 1.31	**5.45E-03**	1458	–	–	–	–
MRS (modified)^a^	1.22	1.10, 1.35	**1.68E-04**	1663	1.15	1.04, 1.27	**6.65E-03**	1852

DNAm-based predictor	T2D in F4 subsample				T2D in FF4 subsample			
	OR	95% CI	*p*	*n*	OR	95% CI	*p*	n
HorvathAA	1.03	0.99, 1.07	0.10	1709	1.00	0.97, 1.05	0.54	1866
HannumAA	1.03	1.00, 1.06	**0.02**	1709	1.00	0.96, 1.04	0.24	1866
GrimAA	1.09	1.04, 1.13	**1.38E-04**	1709	1.09	1.04, 1.14	**2.66E-04**	1866
PhenoAA	1.04	1.01, 1.06	**9.82E-04**	1709	1.03	1.00, 1.06	**0.01**	1866
IEAA	1.03	0.99, 1.07	0.13	1709	1.00	0.96, 1.04	0.97	1866
EEAA	1.03	1.00, 1.05	**0.02**	1709	1.01	0.98, 1.05	0.49	1866
MRS^a^	1.41	1.16, 1.71	**4.47E-04**	1458	–	–	–	–
MRS (modified)^a^	1.41	1.18, 1.68	**1.15E-04**	1664	1.27	1.06, 1.53	**9.83E-03**	1866

Odd Ratios were generated using logistic regression, except for MetS Score using ordinal regression. Models were fully adjusted for chronological age, sex, physical activity level, smoking status, body mass index (only for T2D), alcohol consumption and technical covariates. Regressions of each metabolic condition were based on *N* ≤ 1722 and *N* ≤ 1872 observations in F4 and FF4 subsample respectively, with varying number of observations due to missing values (column “*n*” indicates the number of observations included in each regression under complete case analysis)

MetS, metabolic syndrome; OR, odd ratio; CI, confidence interval; AA, age acceleration; IEAA, intrinsic epigenetic age acceleration; EEAA, extrinsic epigenetic age acceleration; MRS, mortality risk score; T2D, type 2 diabetes

Shown in bold are statistically significant associations (*p* < 0.05)

^a^The coefficients of MRS & MRS (modified) were standardized to its standard deviation

For the regression analysis of MetS score, the Brant test on each of the DNAm-based predictors indicated no violation of the proportional odds assumption. In the sensitivity analysis removing outliers using the Tukey’s 1.5 IQR rule, the observed significant associations in the three aforementioned DNAm-based predictors remained unchanged (see Additional file 2: Table S1–S3 for all sensitivity analysis results). Further adjustment for leukocyte count did not attenuate the associations observed in GrimAA and PhenoAA difference, except for the association between PhenoAA difference and T2D in FF4.

It is noteworthy that the modified version of MRS, which was computed using only eight out of the ten CpGs in MRS, reported a comparable (if not larger) effect size and statistical significance in association with metabolic conditions at F4 as the original MRS. The modified version had two less CpGs which were not present in the EPIC850K BeadChip used at FF4.

### Association of GrimAge components with metabolic conditions

With the concordantly significant associations demonstrated by GrimAA difference across the metabolic conditions, we examined further each of the eight GrimAge components. Of the eight, all components, except for cystatin C, growth differentiation factor 15, and smoking pack-years, reported a positive and statistically significant association with MetS at F4 and FF4 (Additional file 2: Table S4). Plasminogen-activation inhibition 1 (DNAmPAI1) reported the strongest association with MetS consistently at F4 and FF4 (*p* = 1.1E–27, 9.5E–34, respectively). Additionally, DNAmPA1 was the only GrimAge component to demonstrate a significant association with T2D in both F4 and FF4 (*p* = 2.4E–08, 4.8E–14, respectively).

### Longitudinal analysis of time-to-incident T2D

Of the 1456 participants without baseline T2D at S4, 196 (13.5%) developed T2D over the follow-up period. Incidence rate was 9.7 per 1000-person-years [95% CI 8.3–11.1], with median follow-up time of 15.6 years [IQR 13.7–16.0]. Among the 196 participants who were then diagnosed with incident T2D, median time-to-incidence was 7.1 years [IQR 3.8, 11.3]. In the remaining censored participants, one participant developed another diabetes type, 898 remained non-diabetic by the end of the last follow-up, while 361 were lost to follow-up.

**Fig. 1:**
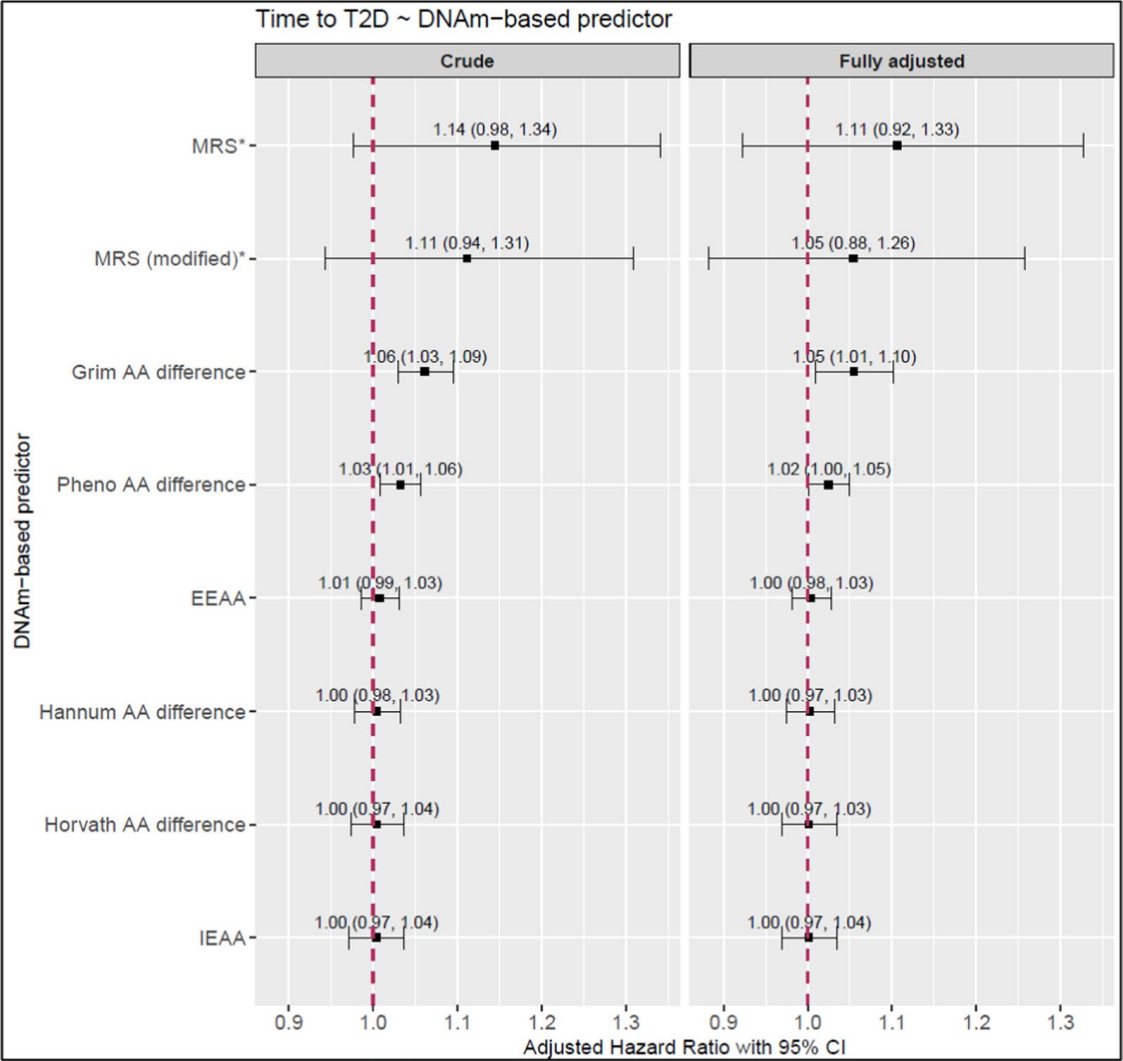
Association of DNAm-based predictors with time-to-incident T2D. Scatter plot showing the effect estimates generated using Cox proportional hazards model under the crude and fully adjusted models. The former adjusted for age, sex and technical covariates, the latter included additionally body mass index, physical activity, smoking status, and alcohol consumption. Abbreviations: T2D, type 2 diabetes; MRS, mortality risk score; AA, age acceleration; EEAA, extrinsic epigenetic age acceleration; IEAA, intrinsic epigenetic age acceleration. *Effect estimate standardized to one standard deviation of the DNAm-based predictor

Of the seven DNAm-based predictors, GrimAA and PhenoAA difference were significantly associated with time-to-incidence of T2D in both crude and fully adjusted models (Fig. 1). One-year increase in GrimAA and PhenoAA difference was significantly associated with an increased hazard of 5.5% [1.0–10.2%] and 2.5% [0.1–4.9%] of developing incident T2D, respectively, in the fully adjusted model. However, both were no longer significant after adjustment for leukocyte count. Upon removal of outliers, only PhenoAA difference remained significant (Additional file 2: Table S5).

### Prognostic value for 8-year T2D incidence risk

Of the 1456 participants without T2D at baseline (S4), those aged ≤ 54 years (*n* = 818) had no measurement of fasting glucose and/or lipid levels and were excluded from the AUC analysis. Additionally, 24 and 133 participants were further excluded due to fasting glucose level above 126 mg/dL (which was indicative of T2D) and missing values for BP or parental history of T2D, respectively. The final sample included 481 participants, out of whom 31 participants were newly diagnosed with T2D by the 8th year.

**Fig. 2:**
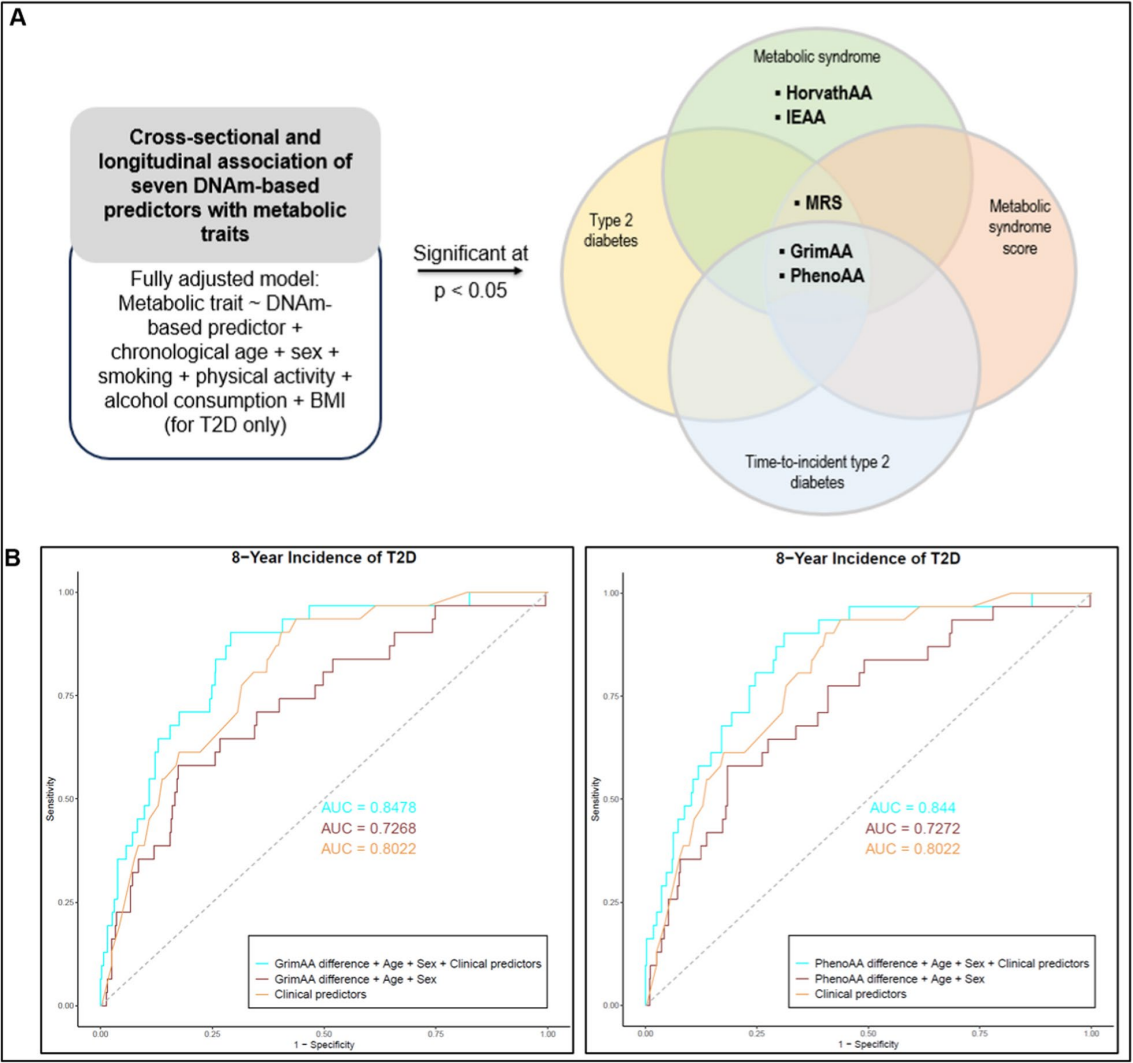
Prognostic value for 8-year T2D incidence risk using DNAm-based predictors which were consistently significant in the preceding analyses using both F4 and FF4 subsample. The Venn diagram (Panel A) shows the predictors significant at *p* < 0.05 under the fully adjusted model for the respective outcome. Panel B (left) illustrates the receiver-operating-characteristic curves of the (i) model comprising the clinical predictors from the Framingham 8-year T2D risk function**,** (ii) model including GrimAA difference, age and sex additionally, and (iii) model with GrimAA difference, age and sex (without Framingham clinical predictors). Panel B (right) illustrates the corresponding curves with PhenoAA difference. AUC indicates the *C*-statistic value. Abbreviations: BMI, body mass index; T2D, type 2 diabetes; AA, age acceleration; IEAA, intrinsic epigenetic age acceleration; MRS, mortality risk score; AUC, area under the curve

In both cross-sectional and longitudinal analyses, GrimAA and PhenoAA difference showed consistently significant associations (Fig. 2A). Accordingly, we compared the AUC between the model fitted only with clinical covariates, comprising predictors from the Framingham 8-year T2D risk function, with models fitted with the DNAm-based predictors. When comparing the model with the Framingham clinical predictors to the model with GrimAA or PhenoAA difference (adjusted for age and sex), both models showed comparable discriminative ability, as evidenced by the overlapping receiver-operating-characteristic (ROC) curves (Fig. 2B). With the inclusion of DNAm-based predictor to the model with Framingham clinical predictors, the discriminative ability improved, though not significantly. For example, after adding GrimAA difference, the C-statistic increased from 0.8 [0.7–0.9] to 0.9 [95% CI 0.8–0.9] in the model with Framingham clinical predictors alone.

As for the model fit of MRS, which was significantly associated with metabolic conditions in cross-sectional analyses, the model’s discriminative ability similarly improved, though not significantly, upon adding MRS to the model with Framingham clinical predictors (Additional file 2: Table S7). Conversely, adding two DNAm-based predictors, instead of only one, did not improve model fit, as depicted by the overlapped ROC curves (see Additional file 2: Figure S5).

## Discussion

Of the seven DNAm-based predictors, GrimAA and PhenoAA difference were identified to be consistently significant in their associations with prevalent MetS and T2D as well as incident T2D, independent of established risk factors. MRS and the modified version showed significant associations with the metabolic conditions cross-sectionally, but not longitudinally with incident T2D. Lastly, the DNAm-based predictors demonstrated largely comparable prognostic values for the 8-year risk of developing T2D as the model with Framingham clinical predictors alone. While adding the DNAm-based predictor to the clinical model improved the model’s discriminative ability, the improvement was not significant. Our findings support the literature in the following aspects: (i) existing DNAm-based predictors presented differential association with diseases, with the second-generation epigenetic measures to be more closely related with metabolic conditions than the first-generation measures; (ii) in predicting incident T2D, DNAm-based predictors might contribute additional prognostic value.

This study sought to compare the utility of the various DNAm-based predictors, in line with the postulation that each of them, with its respective CpGs coverage, represents different aspects of biological aging, despite some biological similarities in terms of overlapping genomic locations [17]. Epigenetic measures have reported varying associations with health outcomes in the literature, which was similarly reported in this study. Unsurprisingly, all DNA-based predictors showed poor correlation with each other, except for the two pairs of related measures, as observed in other studies [12, 14, 15, 25]. Overall, the observed patterns suggest that metabolic conditions were more related to the training phenotypes of MRS, GrimAA and PhenoAA difference, namely age correlates of all-cause/aging-related mortality, as compared to chronological age used in the first-generation clocks.

### Association of second-generation epigenetic measures with metabolic conditions

GrimAA difference reported the strongest statistical significance in all analyses among the DNAm-based predictors. This finding concurs with other recent studies, in which GrimAA difference, or its counterparts (GrimAA residual/DNAmGrimAge), outperformed other measures in the association or prediction of metabolic phenotypes [10, 16, 22, 24, 32]. Notably, some of these studies differed in the profile of study participants, with samples from other ancestries, such as East Asian (Korean) and African American populations [16, 22, 24], suggesting generalizability across ancestries. Nevertheless, the longitudinal association observed between GrimAA difference and incident T2D should be interpreted with caution, as it may have been driven by outliers.

The associations between GrimAA and metabolic conditions could be attributed to its constituents, consisting of seven DNAm-estimated plasma proteins and smoking pack-years [10]. Similar to other studies, DNAm-plasminogen activator inhibitor-1 (PAI1) appeared to predominantly drive the associations, given that it reported the smallest p-value among the eight components [10, 22]. Lu et al., who developed the GrimAge clock, found that DNAm-PAI1 outperformed other components and even GrimAA in the association with metabolic conditions [10]. Growing evidence indicated that the relationship could be bi-directional. In vitro studies highlighted that glucose and triglycerides, stimulated the expression of PAI-1 [32, 33]. Conversely, elevated PAI-1 level could impair insulin clearance as well as promote insulin resistance, thrombosis, and fibrosis, consequently resulting in the development of MetS, T2D and macrovascular complications [34–36]. As for adrenomedullin (ADM), the second most significantly associated GrimAge component after PAI-1, its positive associations with the metabolic conditions are consistent with its physiological effects. Increased ADM level has been linked to acute hyperinsulinemia, oxidative stress, and endothelial injury, contributing to diabetic complications [37].

Compared to GrimAA, PhenoAA in relationship to metabolic traits/diseases was less frequently studied in the literature. The significant associations of PhenoAA with MetS and T2D in this study concur with the findings from Levine et al.’s large-scale study, which reported significant positive correlations between PhenoAA and all metabolic components, including glucose, triglycerides and HDL-C [9]. On the other hand, in a different study cohort of African Americans, PhenoAA was found to be significantly associated with glucose but not with lipid traits [16].

After adjusting for leukocyte count, the significant associations observed in GrimAA and PhenoAA difference remained largely unattenuated. This suggests that PhenoAA and GrimAA difference, as extrinsic measures of aging, capture not only immunosenescence processes but also intrinsic epigenetic changes. Conversely, EEAA, a measure of immune system aging, was not significant in most of the examined associations. This is also reflected in the study by Nannini et al., which postulated that cell-intrinsic aging plays a larger role than immunosenescence in MetS [21].

To our knowledge, existing research has focused only on the association of MRS with metabolic phenotypes other than MetS and T2D. For example, MRS, as a categorical variable, was significantly associated with time to cardiovascular-related mortality in the KORA cohort, which was not reported in other measures, such as HorvathAA and HannumAA [11]. In the same study cohort, we observed that MRS was significantly associated with prevalent metabolic conditions. Notably, the modified version of MRS remained as robust a predictor as the original in our study. The previous methylation array (Infinium HumanMethylation450K) was no longer commercially available, however the modified version based on the new EPIC array, which is missing CpG probes cg06126421 and cg23665802, has demonstrated similar associations. This suggests that the two missing CpGs are likely less relevant to metabolic conditions. Similarly, the DNAmAgeHorvath and DNAmAgeHannum clocks, using the EPIC array, have several missing CpGs; however, McEwen et al. concluded that this difference did not affect their utility [38].

While this study does not aim to ascertain the biological mechanisms, one possible pathway underlying the observed associations with metabolic conditions is the mediating role of aging-related sterile proinflammatory mechanisms, a condition coined as “inflammaging”, which drives various chronic disease phenotypes [39]. It has been proposed that MRS tracks effects of oxidative stress and the resulting systematic inflammation, given its robust association with oxidative stress markers [13, 39]; while PhenoAge has been associated with the activation of proinflammatory pathways, such as interferon signaling [9]. As for GrimAge, several genomic locations of its CpGs have been implicated in inflammaging, including the cytokine-mediated signaling pathway and the response to interferon-gamma [10, 39, 40]. However, the underlying inflammaging-pathways may be unique for each DNAm-based predictor, as there is no overlap between the 513 CpGs in PhenoAge and the 10 in MRS [10, 11]. Overlap with the CpGs in GrimAge could not be determined since the list has not been published.

Overall, we observed a clear pattern of significant associations between metabolic conditions and second-generation epigenetic measures, despite the mixed evidence in the literature regarding associations with first-generation measures. This could be partly attributed to the different study samples. For example, Nannini et al. observed positive associations between MetS score and both IEAA and EEAA among participants of European and African American ancestry (while our study consisted solely of Europeans) [21]. In fact, it has been shown that African Americans had lower EEAA than Europeans [7]. Grant et al. found a positive association between HorvathAA difference and fasting glucose; however, the study participants were post-menopausal women while our sample was from the general adult population [23].

### Study implications

As elucidated earlier, comparisons across existing studies and interpretation of the varying findings on the association with metabolic traits/diseases are not straightforward due to the different profile of study participants, covariates for adjustment, and disease endpoints. Given the inconclusive evidence on DNAm-based predictors for metabolic conditions, future research should focus on replication in other independent cohorts, trans-ancestry meta-analyses, and ancestry-specific studies to account for methylation differences across ancestries.

Additionally, studies using methods such as Mendelian randomization are needed to examine causality. While we prospectively examined baseline methylation in relation to disease incidence, we did not determine whether DNAm drives aging leading to aging-related diseases like T2D, or simply serves as a surrogate marker for early-stage disease methylation variation. The largest genome-wide association study to date, analyzing epigenetic measures across 150 traits using Mendelian randomization, reported a causal effect of BMI and waist circumference on increased GrimAA and PhenoAA but no effects of epigenetic measures on T2D or vice-versa [41]. This suggests a possible mediating role of DNAm-based predictors in metabolic conditions. Should DNAm changes at the CpGs of these predictors be shown to mediate or induce metabolic conditions, they could provide insights into potential therapeutic targets for preventing or treating the disease.

Lu et al. published an updated version of DNAmGrimAge (version 2), which included two additional DNAm-estimated plasma proteins: C-reactive protein and hemoglobin A1c [42]. The AA measure of DNAmGrimAge version 2 reported stronger associations with several age-related conditions and time-to-incidence of cardiovascular diseases, as compared to the original version [42]. Future research is needed to explore the relationship between GrimAA version 2 and metabolic conditions in the European population, particularly given the significant associations of GrimAA difference observed in our study.

Our findings demonstrated the comparable utility of DNAm-based predictors as clinical predictors in predicting the risk of T2D. GrimAA and PhenoAA difference, measured as the extent of divergence from chronological age, appeared to capture an aspect of aging in the development of metabolic conditions which was not reflected in chronological age alone. This is further supported by the lack of correlation between the DNAm-based predictors and chronological age, as reported similarly in other studies [14, 23, 25]. In light of their potential prognostic value for T2D incidence, further research is warranted to explore their clinical utility as biomarkers for risk stratification and prognosis of chronic diseases.

### Study strengths and limitations

Strengths include the prospective nature of the study with a long duration of follow-up to complement the cross-sectional analyses to reflect the associated risk with prevalent and incident metabolic conditions. Findings are based on the KORA cohort, which is a well-characterized prospective study while the participants are largely representative of the general population of European ancestry. Our findings portrayed a consistent pattern of associations observed for the second-generation clocks, highlighting their relevance to metabolic conditions, with potentially some shared biological mechanisms.

A key limitation is the small sample size and the relative low number of events in the AUC analyses, since the majority had no measurement of one or more of the predictors required in the Framingham 8-year T2D risk function. To prevent overfitting, we included the batch variable and the first five PCs, instead of 20 PCs used in the other analyses. Nevertheless, the AUC analyses lacked adequate power, resulting possibly in the insignificant improvement observed in the models added with DNAm-based predictors.

Overall, residual confounding after adjustment for cell type confounding and technical effects cannot be ruled out. We observed that most of the significant associations were not fully attenuated after adjusting for leukocyte count, indicating that the relationships were not merely spurious associations between the metabolic conditions and cell type proportions. We have also adjusted for batch effects, which may arise due to the processing of DNAm across different timepoints and array types used.

Of note, studies have highlighted the unreliability of these epigenetic clocks due to technical noise, particularly compromising longitudinal tracking of epigenetic age [43]. While our analyses did not assess changes in repeated measures over time, technical variations were unlikely to alter the observed association patterns in our study. Additionally, we repeated all analyses using AA residuals in the fully adjusted model (results not shown here), as AA residuals have been proposed as more robust than AA difference in accounting for these technical effects [38], and we did not observe any notable differences. Nonetheless, given this limitation, future longitudinal research should leverage the improved reliability of PC-based epigenetic clocks for more robust results [43]. Conversely, when integrating epigenetic markers into clinical screening, the inefficiency of PC clocks should be considered, as this approach requires substantially more CpGs than the traditional method to generate results.

Another limitation is the risk of informative censoring among participants lost to follow-up, particularly if they were at a higher risk of dying or withdrawing from the study due to worse health conditions, including the development of T2D. Compared to participants who remained in the study cohort until the last follow-up (FF4), both groups differed significantly in age and several health and lifestyle factors. However, the differential distribution of these characteristics likely had no impact on the overall association patterns, as we used the same study sample to compare associations across the seven DNAm-based predictors. Lastly, we did not adjust for multiple testing for the multiple analyses conducted across seven DNAm-based predictors, given the exploratory nature of the study for hypothesis-generating purposes.

## Conclusions

In evaluating the cross-sectional and longitudinal associations between the seven DNAm-based predictors with metabolic conditions (MetS and T2D), we identified a concordant positive association for GrimAA and PhenoAA difference, indicating that a higher AA difference is linked to increased odds of prevalent metabolic conditions and higher risk of incident T2D. MRS and its modified version were found to be robustly and positively associated with both conditions cross-sectionally (but not in longitudinal analysis). These DNAm-based predictors showed comparable discriminative ability to the Framingham clinical predictors and, when added to the model, improved the prediction of 8-year incident T2D, though not significantly.

Overall, our findings are in line with multiple studies on the associations between metabolic conditions and the DNAm-based predictors. These three epigenetic measures from the second-generation clocks likely capture some of the biological variability underlying aging-related diseases, making them potentially valuable biomarkers for risk stratification and disease progression prognosis. Although we did not examine the underlying biological mechanisms, our findings support the hypothesis that inflammaging, cellular senescence and, to a lesser extent, immune system aging, are implicated in the pathophysiology of epigenetic aging and the development of MetS and T2D. Future research is required to assess the utility and feasibility of incorporating these DNAm-based biomarkers into clinical settings, as well as their generalizability across ethnicities. Additionally, functional analyses of genetic and epigenetic regulation are warranted to understand the complex dynamics between epigenetic aging and disease development.

## Supplementary Information

Supplementary file 1. Figures S1–S5 present the flow chart of thestudy sample, pairwise correlation matrices of the seven DNAm-basedpredictors, and ROC curves.

Supplementary file 2. Tables S1–S7 present the results of all conductedanalyses not shown in the main tables and an overview of the DNAmbasedpredictors examined in this paper.

## Data Availability

The informed consent given by the KORA participants excludes posting of participant-level data in public databases. However, data can be made available upon request from the application digital tool KORA.PASST (https://www.helmholtz-munich.de/epi/research/cohorts/kora-cohort/data-use-and-access-via-korapasst/index.html). The variables lists are available for viewing and download from the website, but online request for data/biosamples is subject to approval by the KORA board.

## Abbreviations

AA: Age acceleration (defined as the difference between chronological age and DNAmAge)
BMI: Body mass index
CI: Confidence interval
CpG: Cytosine-phosphate-guanine dinucleotide
DNAm: DNA methylation
DNAmAge: DNA methylation age (estimated epigenetic age based on DNA methylation values)
EEAA: Extrinsic epigenetic age acceleration
GrimAA: Grim age acceleration
HorvathAA: Horvath age acceleration
HannumAA: Hannum age acceleration
HDL-C: High-density lipoprotein cholesterol
HR: Hazard ratio
IEAA: Intrinsic epigenetic age acceleration
IQR: Interquartile range
KORA: Kooperative Gesundheitsforschung in der Region Augsburg
MetS: Metabolic syndrome
MRS: Mortality risk score
OR: Odds ratio
PhenoAA: Pheno age acceleration
ROC curve: Receiver-operating-characteristic curve
SD: Standard deviation
T2D: Type 2 diabetes
