# Supplementary material for: Cross-sectional and longitudinal association of seven DNAm-based predictors with metabolic syndrome and type 2 diabetes
Source: Clin Epigenetics. 2025 Apr 8;17:58. doi: 10.1186/s13148-025-01862-8 (PMC11978091; doi:10.1186/s13148-025-01862-8)
Supplement: Supplementary file 1 — Figures S1–S5 present the flow chart of thestudy sample, pairwise correlation matrices of the seven DNAm-basedpredictors, and ROC curves. [file 13148_2025_1862_MOESM1_ESM.docx]

**Cross-sectional and longitudinal association of seven DNAm-based predictors with Metabolic Syndrome and Type 2 Diabetes**

**Additional file 1**

Table of Contents

[Additional file 1: Figure S1. Flow chart of the study sample and subsample for the respective analyses. 2](#_Toc177998641)

[Additional file 1: Figure S2. Pearson’s correlation between chronological age and DNA methylation age (measured at baseline S4). 3](#_Toc177998642)

[Additional file 1: Figure S3. Pearson’s correlation between chronological age and the seven DNAm-based predictors (measured at baseline S4). 4](#_Toc177998643)

[Additional file 1: Figure S4. Pearson’s correlation of the seven DNAm-based predictors across the three timepoints. 6](#_Toc177998644)

[Additional file 1: Figure S5. Receiver-operating-characteristic (ROC) curves of clinical model compared to models incorporating additionally DNAm-based predictors. 7](#_Toc177998645)


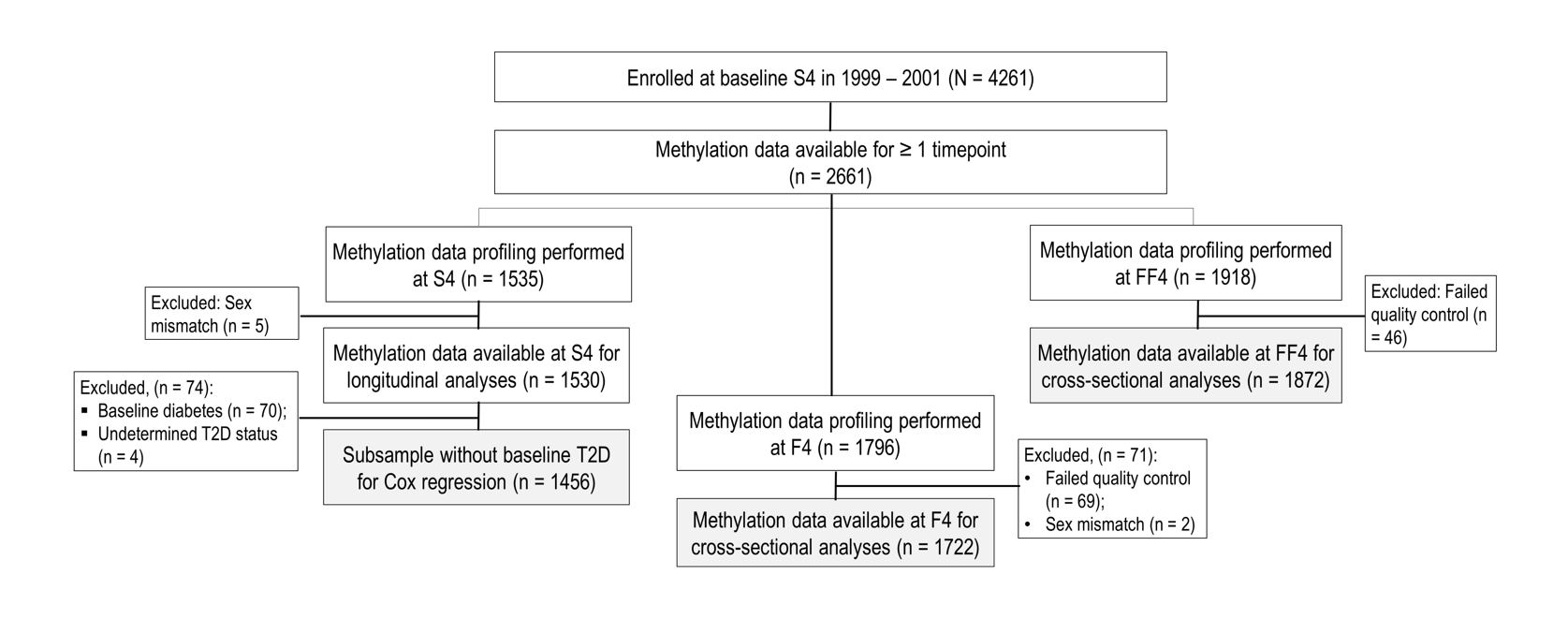


# Additional file 1: Figure S1. Flow chart of the study sample and subsample for the respective analyses.


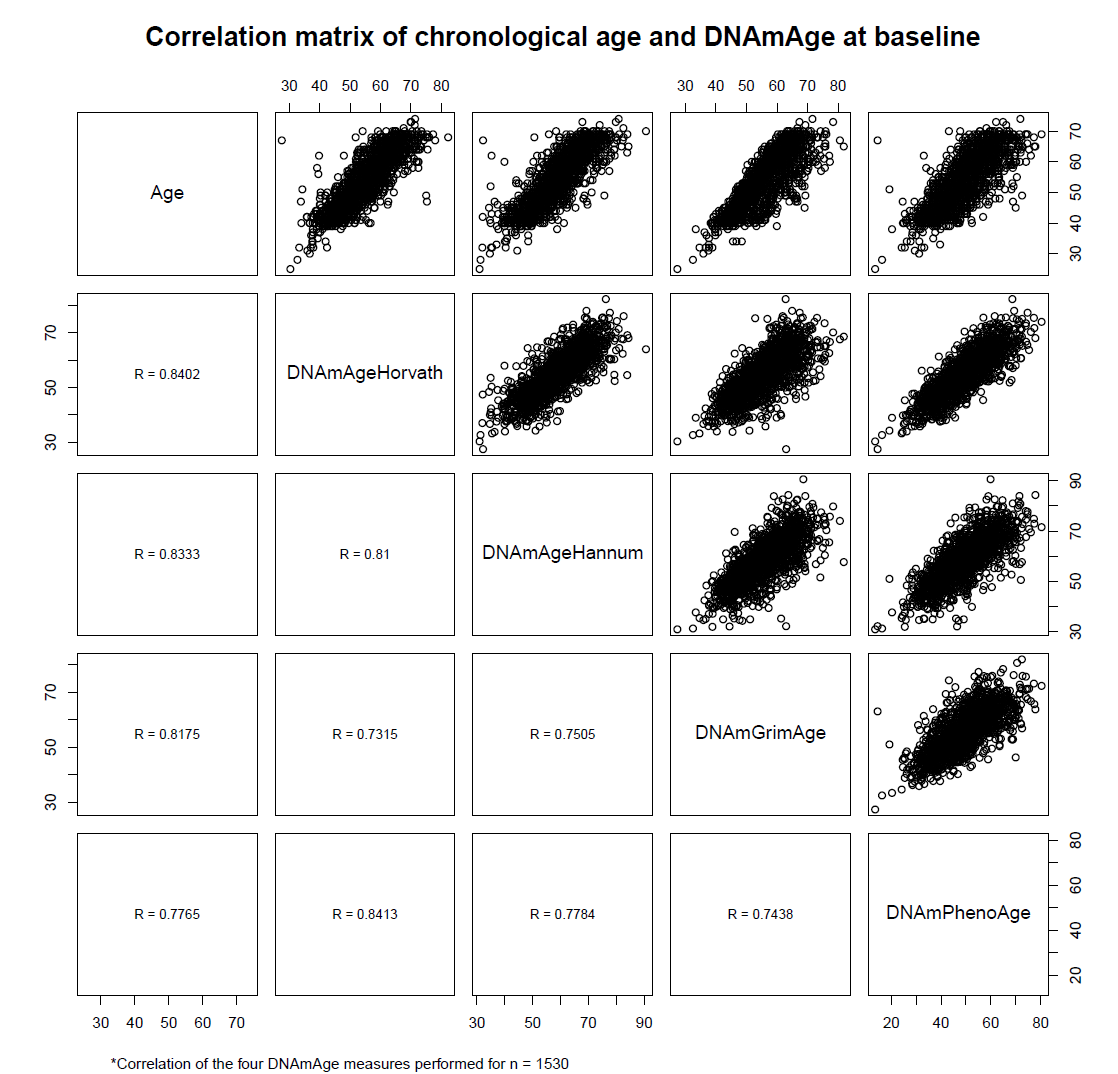


# Additional file 1: Figure S2. Pearson’s correlation between chronological age and DNA methylation age (measured at baseline S4).


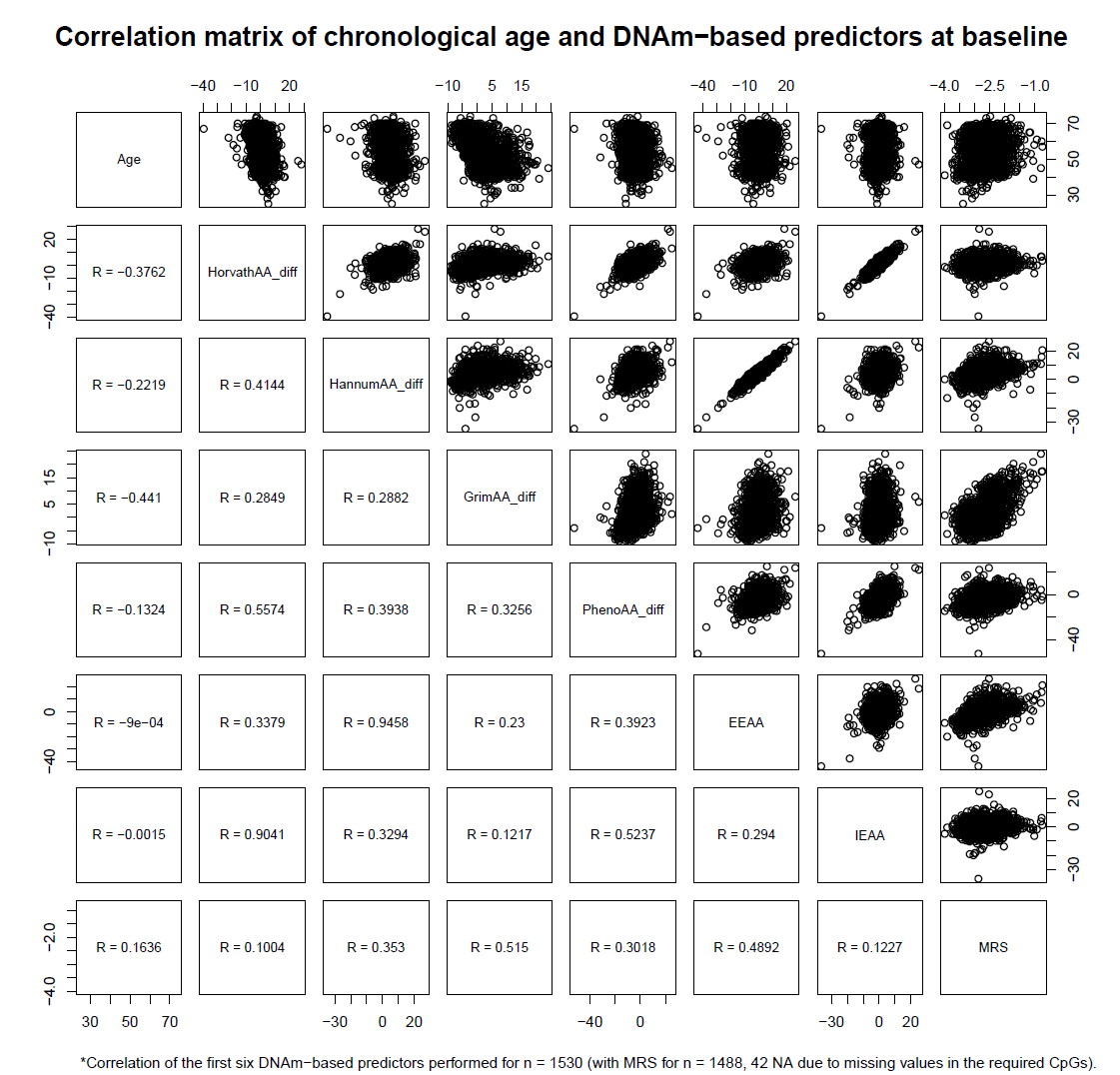


# Additional file 1: Figure S3. Pearson’s correlation between chronological age and the seven DNAm-based predictors (measured at baseline S4).


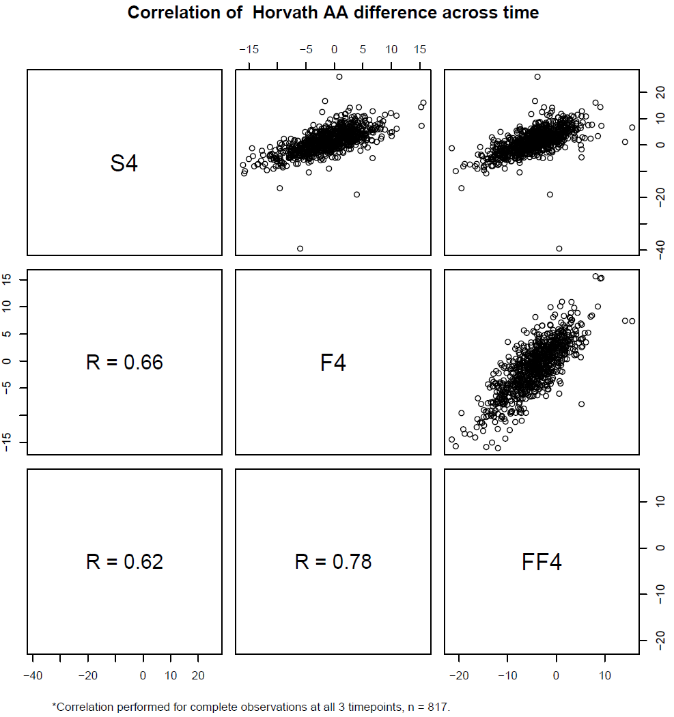

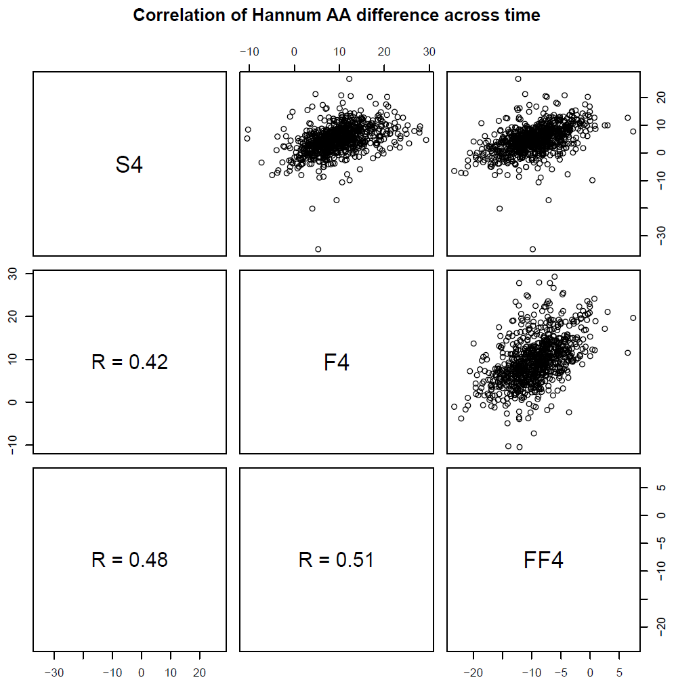
 **A B**


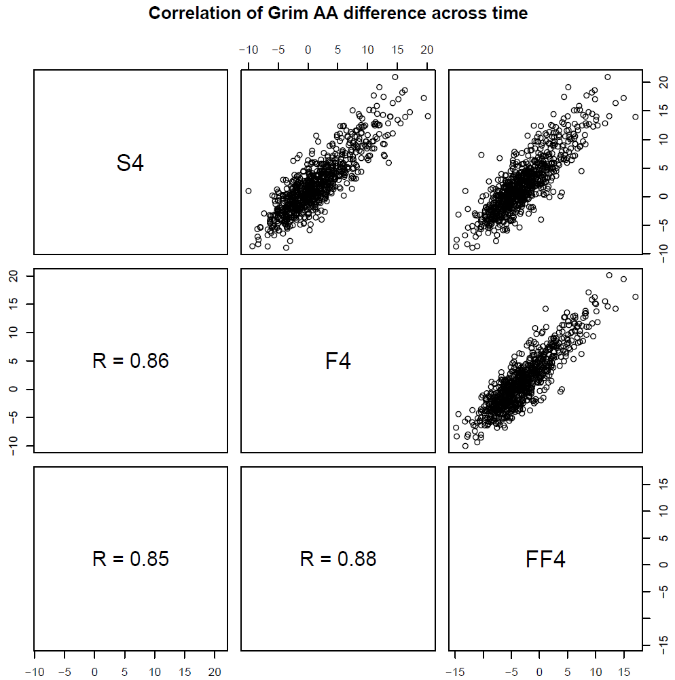


**
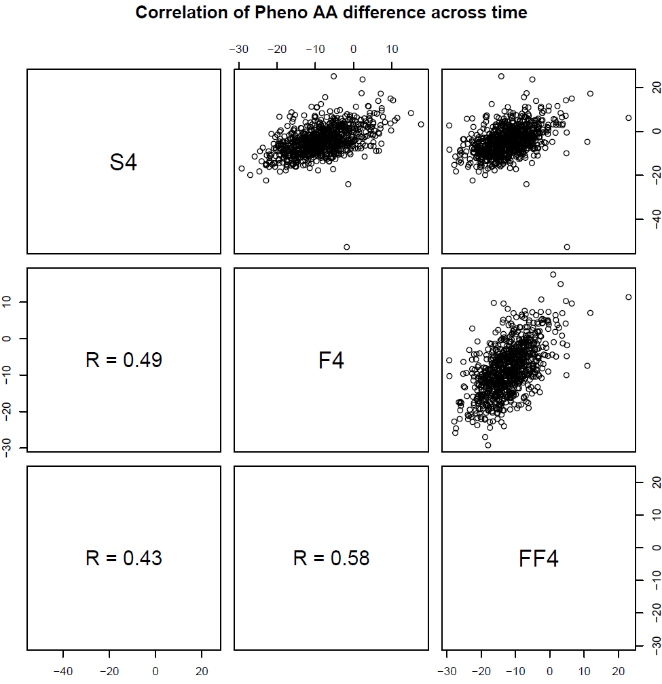
C D**


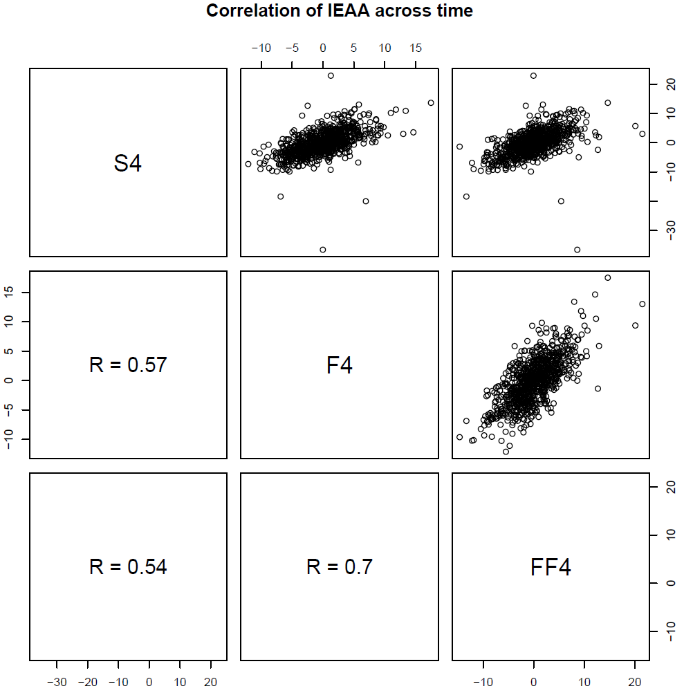
**
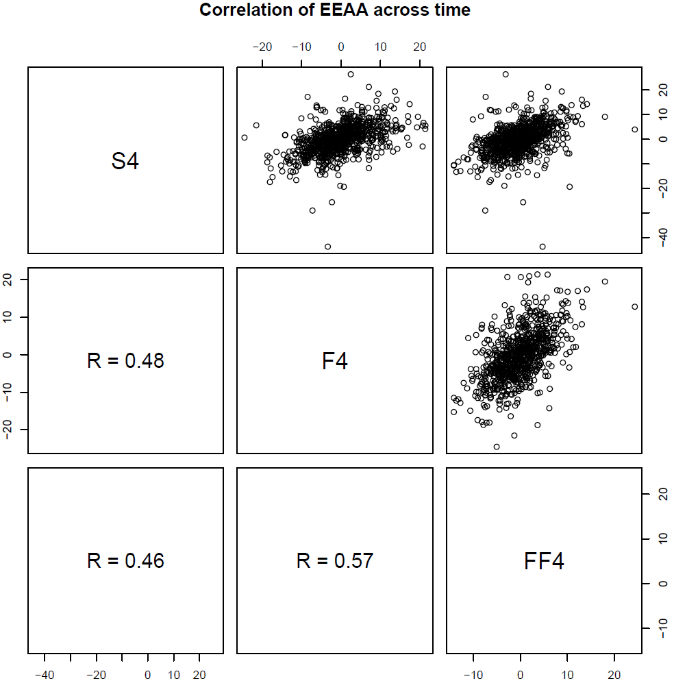
E** **F**


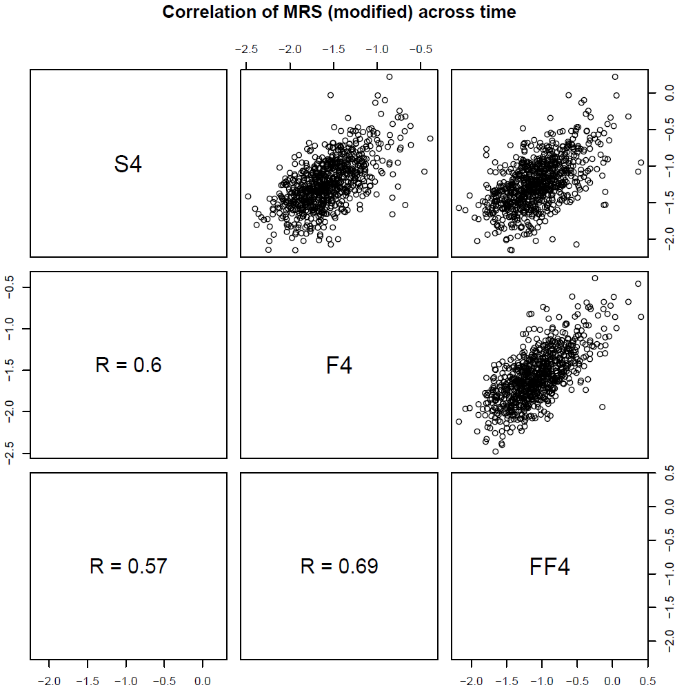
**G**

# Additional file 1: Figure S4. Pearson’s correlation of the seven DNAm-based predictors across the three timepoints.

Panels A-G show the pairwise correlations between each of the three timepoints (S4-F4-FF4) for each of the DNAm-based predictors in the subsample which has three complete observations (n = 817, for the first six measures; n = 783, for MRS (34 NA due to missing values in the required CpGs)).

**A**
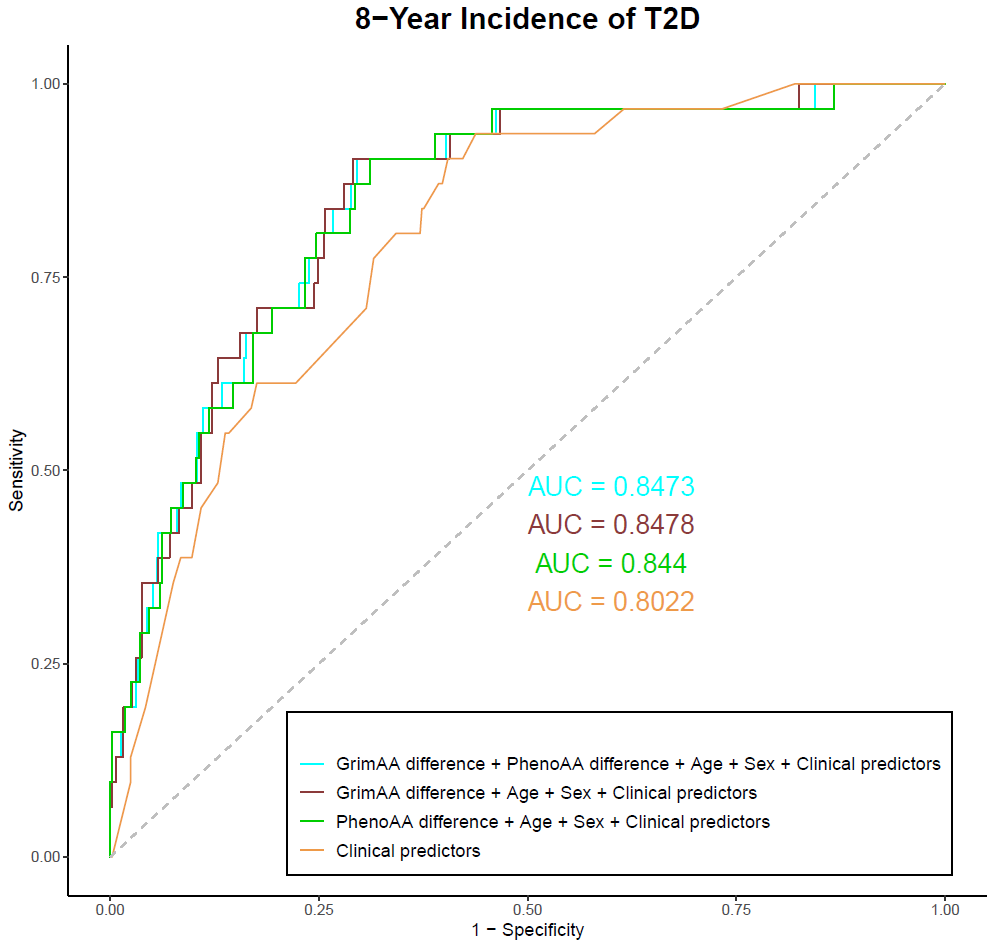
 **B**
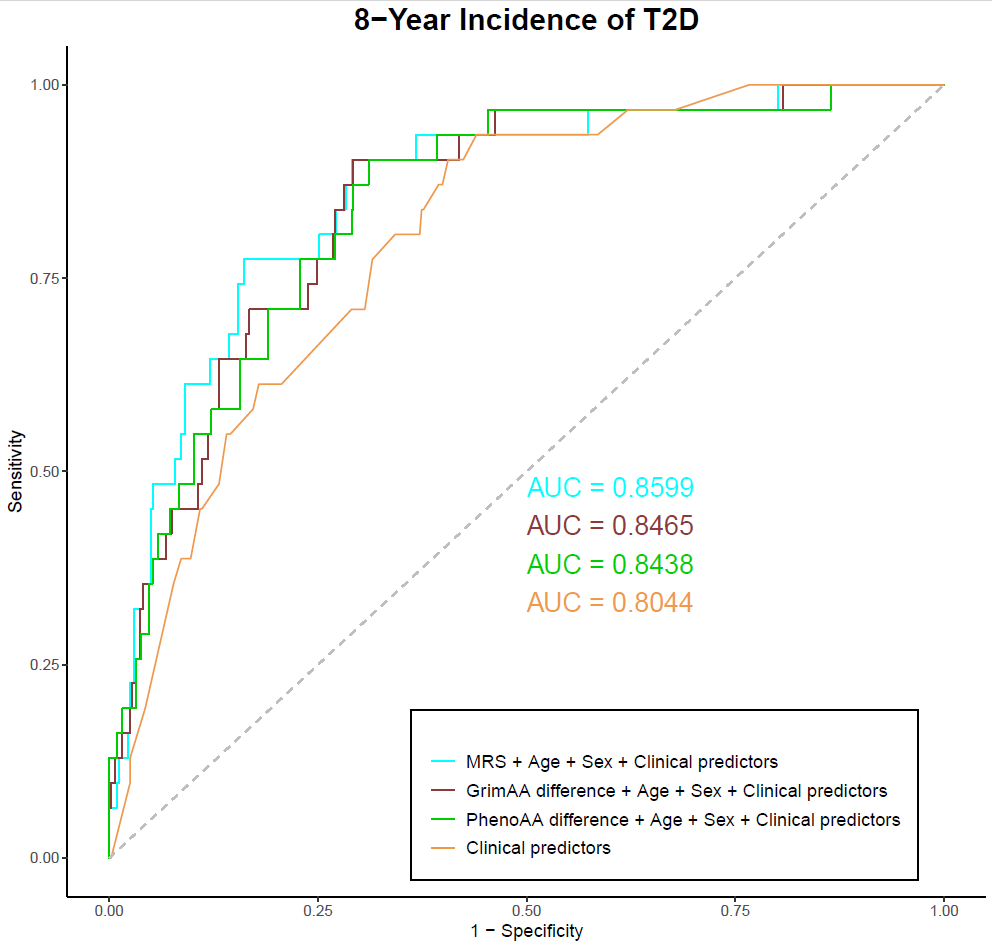


# Additional file 1: Figure S5. Receiver-operating-characteristic (ROC) curves of model with Framingham clinical predictors compared to models incorporating additionally DNAm-based predictors.

Panel A illustrates the curve of model with clinical predictors from the Framingham 8-year T2D risk function versus models including additionally age, sex, GrimAA and/or PhenoAA difference (n = 481). Panel B illustrates the model’s discriminative ability of adding either one of the DNAm-based predictors (MRS, PhenoAA and GrimAA difference) to the model with clinical predictors (n = 472). AUC presented varies slightly (as compared to Panel A) as the analyses used the smaller sample after excluding those with missing MRS values (missing MRS, n = 9). Area under the curve (AUC) indicates the C-statistic value.
